# 72 The role of conservation projects for health enhancing physical activity (HEPA): holistic benefits for volunteers whilst enhancing environmental biodiversity - a blue space case study

**DOI:** 10.1093/eurpub/ckae114.190

**Published:** 2024-09-26

**Authors:** Diane Crone, Faaiza Bashir, Rachel C Sumner, Zsofia Szekeres

**Affiliations:** Cardiff Metropolitan University, Cardiff, Wales, UK; Cardiff University, Cardiff, Wales, UK; Cardiff Metropolitan University, Cardiff, Wales, UK; Cardiff Metropolitan University, Cardiff, Wales, UK

## Abstract

**Purpose:**

This qualitative study explored how engagement activities at a community reservoir in Cardiff, Wales supported the health and wellbeing of the local community, improved access to green and blue spaces, and enhanced the biodiversity of the environment. There were two specific aims; to capture the lived experiences of volunteers involved in the preservation of the reservoir for over 15 years, and; to understand the experiences, motivations, and outcomes of volunteers taking part in community-based conservation activities at the site from 2021. The research is innovative because it captured the experiences of participants (volunteers) who had been involved in the ‘saving of the site’, and volunteers involved in enhancing the site as a community asset during the last 4 years. It therefore captures experiences from nearly two decades of voluntary action in local nature conservation from volunteering perspectives.

**Methods:**

Data were collected through interviews and focus groups from a total of 12 participants (six participants involved in the preservation of the site, and six from the current volunteer base). Data were collected during the summer of 2023 and analysed using thematic analysis (Braun and Clarke, 2019). Analysis provided key findings (themes) linked to the aims detailed above.

**Results:**

Findings from the two sets of volunteers highlighted the following themes: (i) value of nature for health, wellbeing and spirituality; (ii) motivation to taking part in volunteering to support the environment and for self-confidence and a sense of purpose; (iii) health enhancing physical activity opportunities; and (iv) the provision for getting outdoors for activity, fresh air and tranquillity.

**Conclusions:**

Findings highlight the potential of such projects for health enhancing physical activity and the importance of them for holistic mental, social and environmental health benefits for both individuals, and the physical environment. Findings confirm the many ecological, health and wellbeing benefits for both volunteers and the environment from conservation community projects. The holistic benefits of such programmes should be acknowledged, highlighted, and promoted to support both volunteers’ health and to support the current environmental challenges faced in local communities.

